# Novel Insight in Idiopathic Normal Pressure Hydrocephalus (iNPH) Biomarker Discovery in CSF

**DOI:** 10.3390/ijms22158034

**Published:** 2021-07-27

**Authors:** Enrica Torretta, Beatrice Arosio, Pietro Barbacini, Daniele Capitanio, Paolo Dionigi Rossi, Manuela Moriggi, Mario Clerici, Daniela Mari, Matteo Cesari, Cecilia Gelfi

**Affiliations:** 1 IRCCS Istituto Ortopedico Galeazzi, Via Galeazzi 4, 20161 Milan, Italy; enrica.torretta@grupposandonato.it; 2 Department of Clinical Sciences and Community Health, University of Milan, Via Pace 9, 20122 Milan, Italy; beatrice.arosio@unimi.it (B.A.); matteo.cesari@unimi.it (M.C.); 3 Department of Biomedical Sciences for Health, University of Milan, Via F.lli Cervi 93, Segrate, 20090 Milan, Italy; pietro.barbacini@unimi.it (P.B.); daniele.capitanio@unimi.it (D.C.); 4 Fondazione Ca’ Granda, IRCCS Ospedale Maggiore Policlinico, Via F.lli Cervi 93, Segrate, 20090 Milan, Italy; paolo.rossi@policlinico.mi.it; 5 Gastroenterology and Digestive Endoscopy Unit, IRCCS Policlinico San Donato, 20097 Milan, Italy; manuela.moriggi@grupposandonato.it; 6 IRCCS Fondazione Don Carlo Gnocchi, Via Capacelatro 66, 20148 Milan, Italy; mario.clerici@unimi.it; 7 Department of Physiopathology and Transplants, University of Milan, Via Sforza 35, 20122 Milan, Italy; 8 IRCCS Istituto Auxologico Italiano, Via Mercalli 28, 20122 Milan, Italy; daniela.mari@unimi.it; 9 Geriatric Unit, IRCCS Istituti Scientifici Maugeri, Via Camaldoli 64, 20138 Milan, Italy

**Keywords:** idiopathic normal pressure hydrocephalus, sphingolipids, proteome, csf, mass spectrometry

## Abstract

Idiopathic normal pressure hydrocephalus (iNPH) is a potentially reversible neurological disease, causing motor and cognitive dysfunction and dementia. iNPH and Alzheimer’s disease (AD) share similar molecular characteristics, including amyloid deposition, t-tau and p-tau dysregulation; however, the disease is under-diagnosed and under-treated. The aim was to identify a panel of sphingolipids and proteins in CSF to diagnose iNPH at onset compared to aged subjects with cognitive integrity (C) and AD patients by adopting multiple reaction monitoring mass spectrometry (MRM-MS) for sphingolipid quantitative assessment and advanced high-resolution liquid chromatography–tandem mass spectrometry (LC–MS/MS) for proteomic analysis. The results indicated that iNPH are characterized by an increase in very long chains Cer C22:0, Cer C24:0 and Cer C24:1 and of acute-phase proteins, immunoglobulins and complement component fragments. Proteins involved in synaptic signaling, axogenesis, including BACE1, APP, SEZ6L and SEZ6L2; secretory proteins (CHGA, SCG3 and VGF); glycosylation proteins (POMGNT1 and DAG1); and proteins involved in lipid metabolism (APOH and LCAT) were statistically lower in iNPH. In conclusion, at the disease onset, several factors contribute to maintaining cell homeostasis, and the protective role of very long chains sphingolipids counteract overexpression of amyloidogenic and neurotoxic proteins. Monitoring specific very long chain Cers will improve the early diagnosis and can promote patient follow-up.

## 1. Introduction

Despite differences in the disease etiology, idiopathic normal pressure hydrocephalus (iNPH) and Alzheimer’s disease (AD) share cognitive dysfunction as common clinical manifestations [1]. These two disorders have multiple causes, heterogeneous presentations and a different impact on patients. The classic presentation for iNPH is the triad of gait or motor disturbance, cognitive impairment and incontinence [2,3,4]. It is remarkable that AD and iNPH patients present similar molecular characteristics [5], including amyloid deposition [6,7,8] and t-tau and p-tau dysregulation [9]. However, quantitative results of t-tau and p-tau on both iNPH and AD patients alone are not sufficient to monitor different aspects of the neurological damage. In iNPH, symptoms can be partially reversed by cerebrospinal fluid (CSF) clearance, whereas there are not consolidated treatments for other types of dementia. However, despite the improvement of symptoms by shunt surgery, iNPH is under-diagnosed and under-treated. A recent study by Jeppsson A. et al. [10] indicates that a combination of levels T-tau aβ40, APP and MCP-1 can separate iNPH from cognitive and movement disorders with good diagnostic sensitivity and specificity. An enlargement of the panel of molecules targeting different areas such as amyloid β (aβ) production and aggregation, cortical neuronal damage, tau pathology, axonal damage, astrocyte activation and lipid raft would define iNPH patients more precisely.

Sphingolipids are bioactive lipids and represent the major components of the plasma membrane and act as a modulator of cell to cell interactions. The changes in sphingolipids composition may impact not only the structure of the plasma membrane but may also translate signals inducing quali/quantitative changes in protein composition [11]. It is known that the maintenance of sphingolipid homeostasis is essential to prevent cell death and neurodegeneration [12]. 

Having hypothesized that a different sphingolipid and protein composition of CSF may characterize neurological and neurodegenerative disorders, with the present study, we would like to monitor for the first time the CSF profile of sphingolipids and proteins in aged iNPH and AD patients compared to aged subjects with cognitive integrity (C) by multiple reaction monitoring mass spectrometry (MRM/MS) and liquid chromatography–tandem mass spectrometry (LC–MS/MS). The aim was to find a possible relationship between sphingolipid variation and CSF protein composition to select molecules specific for iNPH diagnosis to implement the panel of biomarkers recently described [10].

In our previous pioneering study [13], based on matrix-assisted laser desorption/ionization (MALDI) profiling of low abundant CSF proteins, the top-down MS approach did not reveal differences at protein level between iNPH and C. Concerning sphingolipids, our previous study [14] was based on untargeted approaches, adopting primuline staining for serum samples and MALDI profiling and LC–MS/MS for CSF samples of AD and iNPH patients. Chains of Cers, SMs and S1P were at variance in AD and iNPH. 

In this study, sphingolipid-specific chains were analyzed in parallel on C and patients enrolled for the proteomic study and results indicated changes in lipid raft and proteins, providing not only hints to understand the pathophysiology of this disorder but also a panel of sphingolipids and proteins as putative markers in iNPH and neurodegeneration.

## 2. Results

### 2.1. Subjects’ General Characteristics and Clinical Parameters Assessment

The participants’ age and median levels of CSF Aβ, tau and p-tau proteins were summarized in Table 1. Gender composition was homogeneous across groups whereas the age range was slightly different, being iNPH patients older compared to aged subjects with cognitive integrity (C) and AD patients (ANOVA on ranks *p*-value < 0.001; Dunn’s *p*-value < 0.05 for iNPH vs. C comparison and iNPH vs. AD comparison). Concerning Aβ levels, in iNPH levels were only slightly decreased compared to C; conversely, lower values were observed in AD patients (one-way ANOVA *p*-value < 0.001; Bonferroni’s *t*-test *p*-value < 0.001 for all comparisons) in agreement with previously reported data [15]. Tau and p-tau statistically increased in AD compared to C and iNPH (ANOVA on ranks *p*-value < 0.001; Dunn’s *p*-value < 0.05 for AD vs. C comparison and AD vs. iNPH comparison).

### 2.2. Sphingolipid Profile

**Figure 1:**
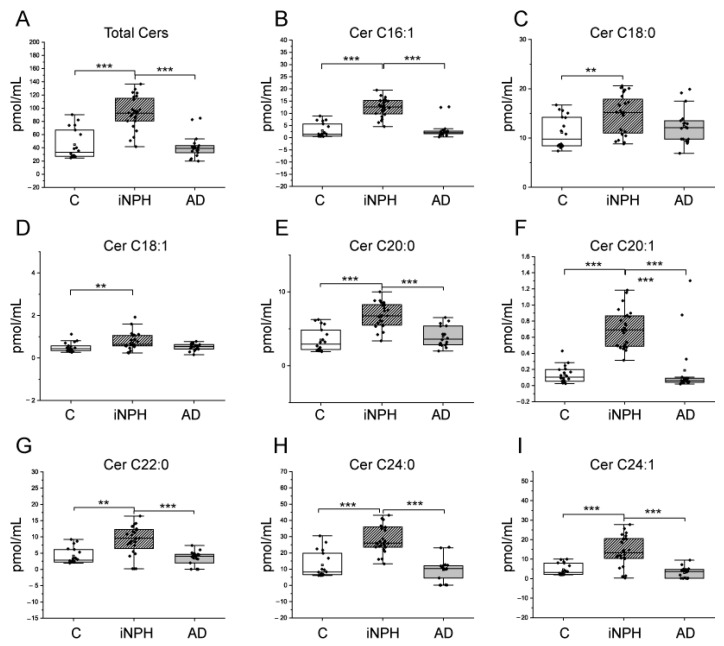
Box plots of CSF ceramide (Cer) species changed in iNPH and AD patients compared to C subjects. (**A**) Total Cers, (**B**) Cer C16:1, (**C**) Cer C18:0, (**D**) Cer C18:1, (**E**) Cer C20:0, (**F**) Cer C20:1, (**G**) Cer C22:0, (**H**) Cer C24:0, (**I**) Cer C24:1. Each measurement was run in triplicate. Data were analyzed using Kruskal–Wallis ANOVA, followed by Dunn’s post hoc test for multiple comparisons. ** *p*-value < 0.01, *** *p*-value < 0.001.

**Figure 2:**
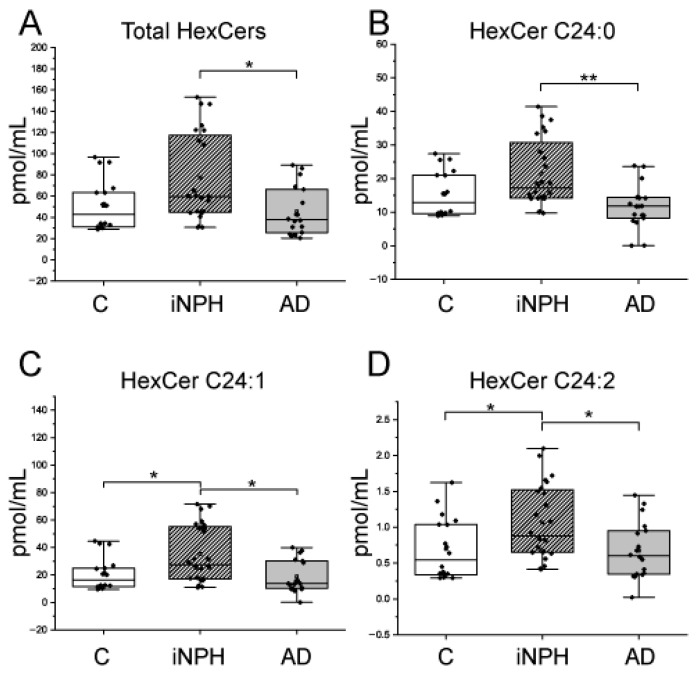
Box plots of CSF hexosylceramide (HexCer) species changed in iNPH and AD patients compared to C subjects. (**A**) Total HexCers, (**B**) HexCer C24:0, (**C**) HexCer C24:1, (**D**) HexCer C24:2. Each measurement was run in triplicate. Data were analyzed using Kruskal–Wallis ANOVA, followed by Dunn’s post hoc test for multiple comparisons. * *p*-value < 0.05, ** *p*-value < 0.01.

Ceramides (Cers), hexosylceramides (HexCers), sphingomyelins (SMs) and dihydrosphingomyelins (DhSMs) CSF levels were investigated by targeted mass spectrometry. Levels of total Cers, Cer C16:1, C20:0, C20:1, C22:0, C24:1 were higher in iNPH patients compared to C and AD patients. Cers C18:0 and C18:1 were significantly higher in INPH compared to C, only (Figure 1). HexCers C24:1 and C24:2 were higher in iNPH compared to C and AD patients, whereas total HexCers and HexCers C24:0 were higher in iNPH compared to AD patients, only (Figure 2).

**Figure 3:**
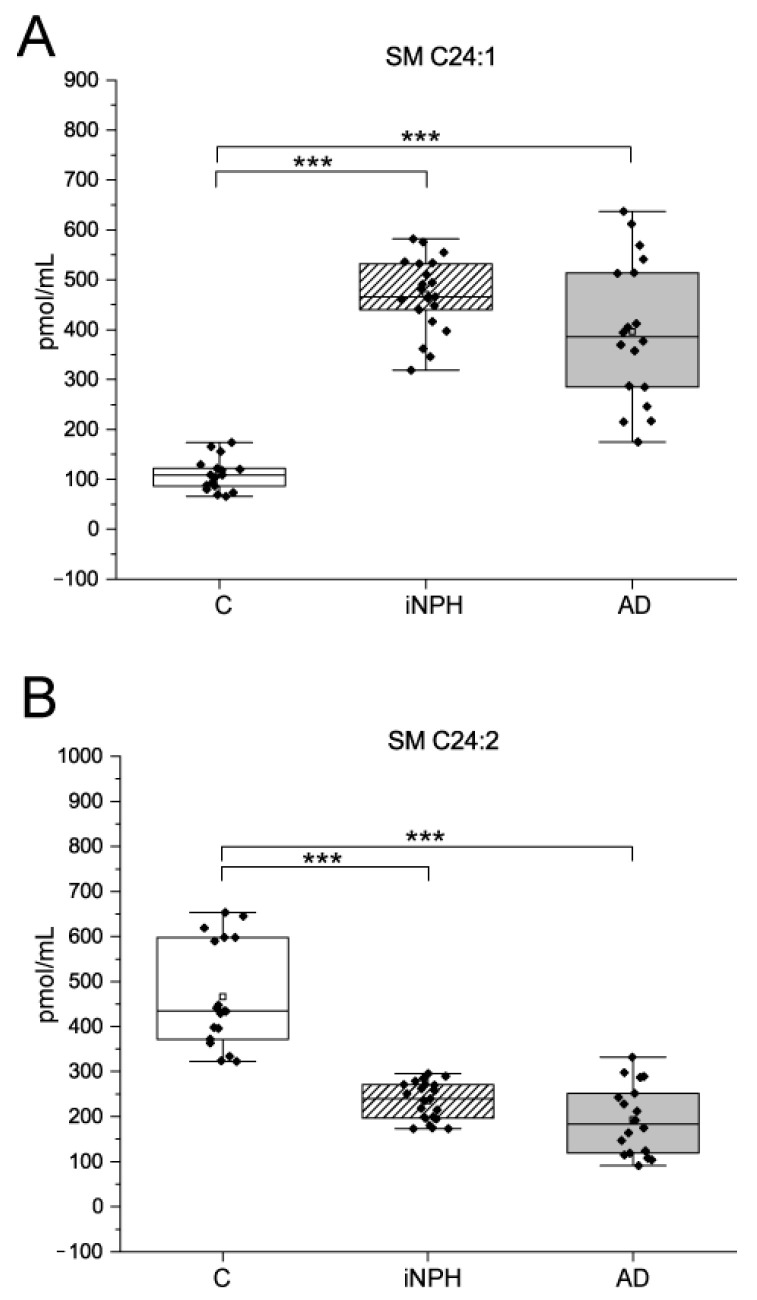
Box plots of (**A**) SM C24:1 and (**B**) SM C24:2 levels in CSF from iNPH and AD patients compared to C subjects. Each measurement was run in triplicate. Data were analyzed using Kruskal–Wallis ANOVA, followed by Dunn’s post hoc test for multiple comparisons. *** *p*-value < 0.001.

Regarding SMs, CSF from iNPH and AD patients showed higher levels of SM C24:1 and lower levels of SM C24:2 compared to C (Figure 3). For all sphingolipids, ROC curves and AUC values were calculated (Table 2). 

### 2.3. Differentially Expressed CSF Proteins in iNPH and in AD Patients Compared to Aged Subjects with Cognitive Integrity

CSF proteome was analyzed by label-free MS to detect statistically changed proteins in the liquor of iNPH or AD patients compared to C.

Label-free LC–MS/MS analyses identified 205 changed CSF proteins in iNPH compared to C and 204 changed proteins in AD compared to C (84 show an opposite trend between iNPH and AD compared to C, whereas 121 show the same trend in iNPH and AD vs. C).

#### 2.3.1. Protein Levels with Opposite Trend in iNPH and AD

**Figure 4:**
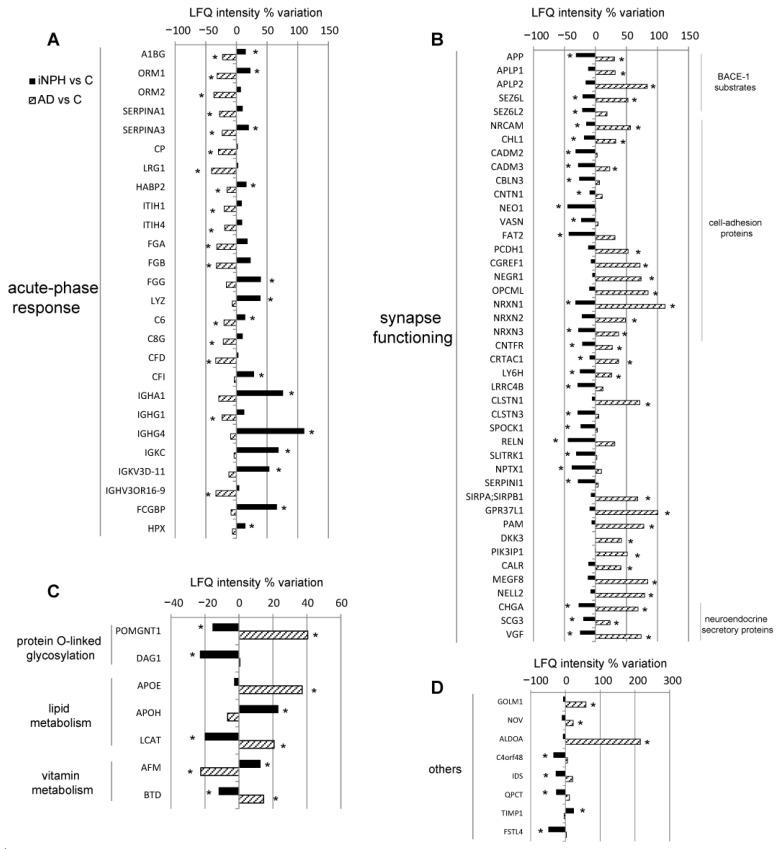
Differentially expressed CSF proteins in iNPH (black bars) and in AD (striped bars) patients compared to healthy cognitive subjects. Histograms of proteins with opposite trend levels in iNPH and AD (LFQ intensity% variation, * *p*-value < 0.05). Proteins were divided into panels according to their function. (**A**) acute phase response proteins. Alpha-1B-glycoprotein (A1BG), alpha-1-acid glycoprotein 1 (ORM1), alpha-1-antichymotrypsin (SERPINA3), hyaluronan binding protein 2 (HABP2), fibrinogen gamma chain (FGG), lysozyme (LYZ) and hemopexin (HPX) complement component C6 and complement factor I (CFI), heavy constant alpha 1 (IGHA1), immunoglobulin heavy constant gamma 4 (IGHG4), immunoglobulin kappa constant (IGKC), immunoglobulin kappa variable 3D-11 (IGKV3D-11) and Fc fragment of IgG binding protein (FCGBP) were increased in iNPH. A1BG, ORM1, SERPINA3, HABP2, C6 levels, as well as alpha-1-acid glycoprotein 2 (ORM2), alpha-1-antitrypsin (SERPINA1), ceruloplasmin (CP), Leucine-rich alpha-2-glycoprotein (LRG1), Inter-alpha-trypsin inhibitor heavy chain H1 (ITIH1), Inter-alpha-trypsin inhibitor heavy chain H4 (ITIH4), fibrinogen alpha chain (FGA), fibrinogen beta chain (FGB), complement component C8 gamma chain (C8G), complement factor D (CFD), Ig gamma-1 chain C region (IGHG1) and Immunoglobulin heavy variable 3/OR16-9 (IGHV3OR16-9) were significantly lower in AD than in C. (**B**) synapse functioning proteins. L1-like protein (CHL1), cell adhesion molecule 2 (CADM2), cell adhesion molecule 3 (CADM3), cerebellin 3 precursor (CBLN3), contactin 1 (CNTN1), neogenin 1 (NEO1), vasorin (VASN), FAT atypical cadherin 2 (FAT2), neurexin-1 (NRXN1), neurexin-3 (NRXN3), ciliary neurotrophic factor receptor subunit alpha (CNTFR), cartilage acidic protein 1 (CRTAC1), lymphocyte antigen 6H (LY6H), leucine-rich repeat containing 4B (LRRC4B), calsyntenin 3 (CLSTN3), testican 1 (SPOCK1), reelin (RELN), SLIT and NTRK Like Family Member 1 (SLITRK1), neuronal pentraxin 1 (NPTX1), neuroserpin (SERPINI1) were decreased in iNPH. Conversely, in AD, levels of NRCAM, CHL1, CADM3, NRXN1 and NRXN3 were upregulated, and increased levels were also observed for protocadherin-1 (PCDH1), cell growth regulator with EF-hand domain protein 1 (CGREF1), neuronal growth regulator 1 (NEGR1), opioid-binding protein/cell adhesion molecule (OPCML) and neurexin-2 (NRXN2). Calsyntenin-1 (CLSTN1), tyrosine-protein phosphatase non-receptor type substrate 1 (SIRPA), prosaposin receptor (GPR37L1), peptidyl-glycine alpha-amidating monooxygenase (PAM), Dickkopf-related protein 3 (DKK3), phosphoinositide-3-kinase-interacting protein 1 (PIK3IP1), calreticulin (CALR), multiple epidermal growth factor-like domains protein 8 (MEGF8), protein kinase C-binding protein (NELL2). (**C**) protein glycosylation, lipid metabolism, vitamin metabolism proteins. Protein O-linked-mannose beta-1,2-N-acetylglucosaminyltransferase 1 (POMGNT1) and Dystroglycan 1 (DAG1) decreased in iNPH. (**D**) other proteins.

With it being our focus the search of new markers to extend the panel of molecules for early diagnosis and follow up of iNPH, we first addressed our attention on proteins with opposite trend between iNPH and AD compared to C (Figure 4 and Supplementary Table S1).

The CSF of iNPH patients was characterized by increased levels of seven proteins of acute-phase response as indicated in the legend of Figure 4A. Moreover, the complement component C6, complement factor I (CFI) and immunoglobulins were increased.

Conversely, proteins belonging to the same class were down-regulated in AD compared to C (Figure 4A).

The proteins active at the synaptic terminals were also at variance, being down-regulated in iNPH compared to C and upregulated in AD compared to C. The substrates of beta-secretase 1 (BACE1), amyloid precursor protein (APP), seizure 6-like protein (SEZ6L) and seizure-related 6 homolog-like 2 (SEZ6L2) were statistically lower in iNPH than in C. Conversely, in AD, APP and SEZ6L levels were statistically upregulated, as amyloid-like protein 1 (APLP1) and amyloid-like protein 2 (APLP2). The same trend was detected for synapse adhesion molecules, for proteins involved in axonogenesis and in signal transduction, and for neuroendocrine secretory proteins as chromogranin-A (CHGA), secretogranin-3 (SCG3) and neurosecretory protein VGF (Figure 4B).

O-glycosylation related proteins were downregulated in iNPH, whereas POMGNT1 was increased in AD liquor (Figure 4C)

Among lipid metabolism, Apolipoprotein E (APOE) increased in AD, whereas apolipoprotein H (APOH) was decreased in iNPH. Lecithin-cholesterol acyltransferase (LCAT) was increased in AD and decreased in iNPH (Figure 4C).

Afamin (AFM) increased in iNPH, whereas it decreased in AD, and biotinidase (BTD) decreased in iNPH and increased in AD (Figure 4C). Other proteins involved in Golgi membrane remodeling and in metabolism increased in AD as Golgi membrane protein 1 (GOLM1), Protein NOV homolog (NOV) and Fructose-bisphosphate aldolase A (ALDOA). Metalloproteinase inhibitor 1 (TIMP1) increased in iNPH, whereas neuropeptide-like protein C4orf48, iduronate 2-sulfatase (IDS), glutaminyl-peptide cyclotransferase (QPCT), follistatin-related protein 4 (FSTL4) were all statistically decreased in iNPH (Figure 4D). For all these proteins, ROC curves and AUC values were calculated (Table 3).

#### 2.3.2. Proteins Levels with the Same Trend in iNPH and AD

**Figure 5:**
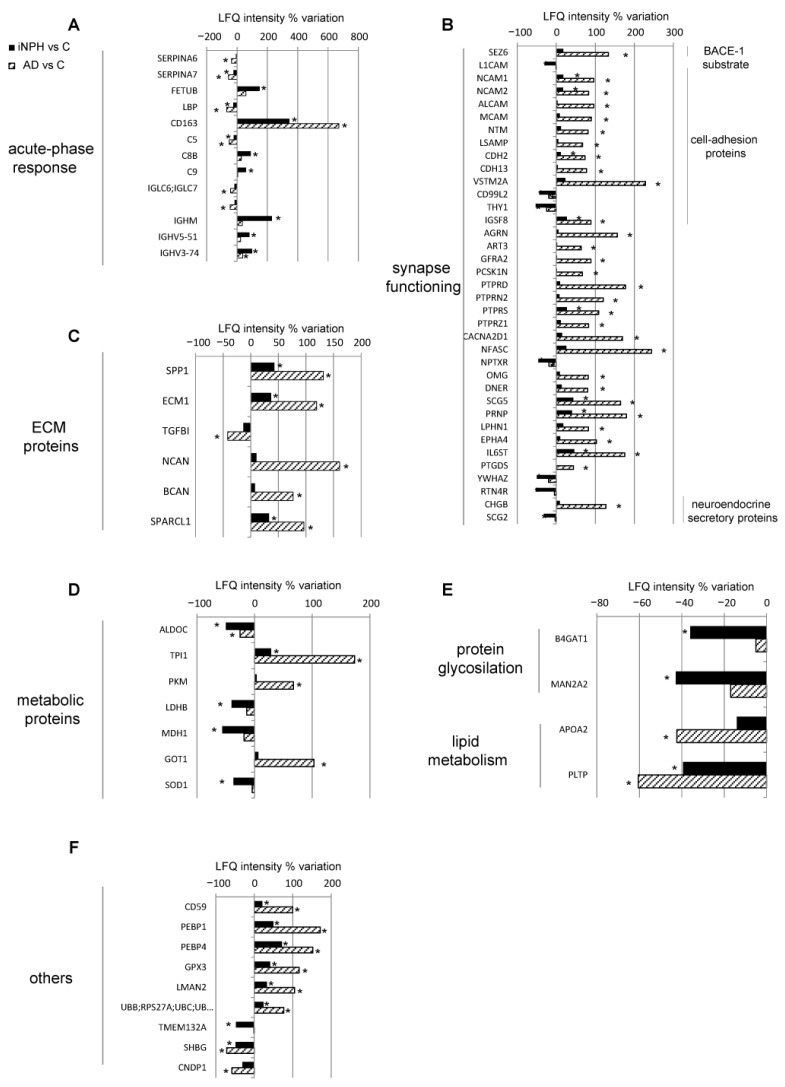
Differentially expressed CSF proteins in iNPH (black bars) and in AD (striped bars) patients compared to healthy cognitive subjects. Histograms of proteins with same trend levels in iNPH and AD (LFQ intensity% variation, * *p*-value < 0.05). Proteins were divided into panels according to their function (**A**) acute phase response (**B**) synapse functioning (**C**) extracellular matrix proteins (**D**) metabolic proteins (**E**) protein glycosylation, lipid transport (**F**) other proteins.

This paragraph examines proteins that follow the same trend in iNPH and AD compared to C with different levels in the two pathologies (difference of average log2 values > 0.5; Figure 5 and Supplementary Table S2).

Among acute phase proteins, the CSF of iNPH patients showed the highest levels of fetuin-B (FETUB), complement component C8 beta chain (C8B), complement component C9, Ig mu chain C region (IGHM), immunoglobulin heavy variable 5-51 (IGHV5-51) and immunoglobulin heavy variable 3-74 (IGHV3-74), whereas scavenger receptor cysteine-rich type 1 protein M130 (CD136) is higher in AD than in iNPH. Moreover, AD showed the lowest levels of corticosteroid-binding globulin (SERPINA6), thyroxine-binding globulin (SERPINA7), lipopolysaccharide-binding protein (LBP), complement C5, Ig lambda-6 chain C region (IGLC6), Ig gamma-2 chain C region (IGHG2; Figure 5A).

The levels of some synaptic proteins in iNPH patients were close to C levels, and they strongly increased in AD, whereas L1 cell adhesion molecule (L1CAM), CD99 antigen-like protein 2 (CD99L2), Thy-1 membrane glycoprotein (THY1), neuronal pentraxin receptor (NPTXR), 14-3-3 protein zeta/delta (YWHAZ), reticulon-4 receptor (RTN4R), secretogranin-2 (SCG2) were more abundant in iNPH than in AD (Figure 5B).

Extracellular matrix proteins and glycoproteins were upregulated mostly in AD. iNPH showed a statistical increase in extracellular matrix proteins (osteopontin (SPP1), extracellular matrix protein 1 (ECM1) and SPARC-like protein 1 (SPARCL1), but not of glycoproteins as neurocan core protein (NCAN) or brevican core protein (BCAN; Figure 5C).

Metabolic proteins as fructose-bisphosphate aldolase C (ALDOC), L-lactate dehydrogenase B chain (LDHB), malate dehydrogenase (MDH1) and superoxide dismutase (SOD1) were downregulated in iNPH. In AD, a statistical decrease was seen only for ALDOC, whereas upregulated levels of triosephosphate isomerase (TPI1), pyruvate kinase (PKM) and aspartate aminotransferase (GOT1) were detected (Figure 5D).

Enzymes involved in glycosylation as beta-1,4-glucuronyltransferase 1 (B4GAT1) and Alpha-mannosidase (MAN2A2) were downregulated in iNPH. 

Lipid transport proteins as apolipoprotein A2 (APOA2) and phospholipid transfer protein (PLTP) were downregulated both in iNPH and in AD, with the lowest levels in AD (Figure 5E).

The changes detected in other proteins were more pronounced in AD, except for transmembrane protein 132A (TMEM132A) that was downregulated in iNPH, whereas its levels in AD were comparable to C (Figure 5F).

### 2.4. IPA Pathway Analysis

**Figure 6:**
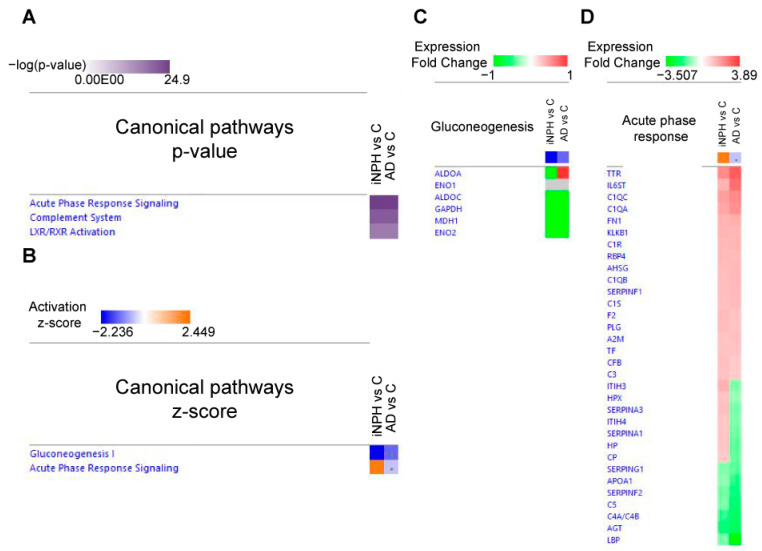
Pathway analysis conducted by IPA software. Canonical pathways were displayed following the *p*-value (**A**) and the z-score (**B**). Shades of purple from dark to light indicate *p*-value values from more to less significant. Orange and blue colors indicate pathway activation (orange; z-score > 2) or inhibition (blue; z-score < 2). Dots indicate non-significant z-score. Gene heatmap of protein expression data in gluconeogenesis pathway (**C**) and acute phase response pathway (**D**) was displayed. In the first pathway, fructose-bisphosphate aldolase C (ALDOC), glyceraldehyde-3-phosphate dehydrogenase (GAPDH), MDH1 and gamma-enolase (ENO2) were decreased both in iNPH and in AD, whereas ALDOA was decreased in iNPH and increased in AD (Figure 3c). In acute phase response, inter-alpha-trypsin inhibitor heavy chain H3 (ITIH3), HPX, SERPINA3, ITIH4, SERPINA1, haptoglobin (HP) and CP resulted in being upregulated in iNPH but not in AD (Figure 3d). Green and red colors indicate a decrease or increase in the protein, respectively. Grey color indicates statistically not significant changes.

The pathway analysis conducted by IPA software indicated that the most significant canonical pathway activated in iNPH was acute phase response signaling (*p*-value 1.08 × 10^−22^; z-score 2.138), not activated in AD (z-score −0.535). The next two significant canonical pathways were complement system (*p*-value 3.41 × 10^−22^; z-score 0.832 both for iNPH and AD) and LXR/RXR that links lipids and metabolism (*p*-value 1.44 × 10^−18^; z-score 1.528 for iNPH and 1.091 for AD; Figure 6A and Supplementary Table S3). The results were filtered by a significant z-score (>2 if the pathway is activated or <2 if the pathway is inhibited), highlighted that the inhibition of gluconeogenesis is more marked in iNPH than in AD (z-scores −2.236 and −1.342, respectively; Figure 6B). The gene heatmap shown in Figure 6C,D showed upregulated and down-regulated proteins in gluconeogenesis and acute phase response pathways.

Two high-scoring networks were generated by IPA: the first (1) grouped 35 proteins mainly involved in neurological disease, scoring 56; the second (2) grouped 35 proteins involved in metabolic disease, scoring 56 (Supplementary Figure S1).

## 3. Discussion

The goal of the studies on neurological disorders is to deliver highly informative bodily fluids set of markers that can promote a differential diagnosis in the early stage of the disease and predict the disease course. In this context, CSF profiling allowed to detect molecules counter-regulated in iNPH vs. AD compared to C and identified a set of them to be monitored to prevent the evolution of iNPH toward AD [16]. The study takes advantage of the adoption of state-of-the-art technologies and the inclusion of C, making the differential protein and sphingolipids abundance more robust compared to a number of studies, including our previous works [13,14].

Sphingolipid levels provided a pattern characteristic of iNPH with increased levels of very long chain Cers, not observed in AD. The studies on animal models indicated that overexpression of very long chains Cers exerts a protective role by improving insulin signal transduction, decreasing ER stress and gluconeogenetic markers [17]. Ceramide synthase 2 null mice that lack Cer C22-24 very long chains displayed defects in the SNC, insulin resistance and biophysical changes in lipid membranes leading to increased ROS production, mitochondrial dysfunction and ER stress. Furthermore, it was observed that the addition of very long chains Cers (C24:0) or CerS2 overexpression in Hep3B hepatic cells showed a protective effect on palmitate-induced ER stress while overexpression of CerS6 coding for Cer C16 has a detrimental effect [18]. However, the contribution of structural changes on membrane properties in patients still requires further studies. Interestingly, in women, very long chains HexCers levels appear to be lower than in men (Supplementary Figure S2), suggesting that a gender difference may exist. Therefore, further studies in this direction can contribute to defining the question of disease prevalence indicated by some studies and better understand the role of glucosylceramides in brain disorders [19,20,21].

The CSF proteome also provides a protein pattern characteristic of iNPH. The latter is characterized by increased acute-phase proteins, immunoglobulins and complement component fragments, indicating a disruption of the BBB barrier and inflammation as a consequence of endothelial leakage and inflammatory cell infiltration [22]. In a healthy brain, the local production of complement regulators is low, whereas complement receptors are expressed on glia and neurons possibly to respond to local complement activation [23]. In iNPH, C1 is involved in a cascade of sequential events leading to the increment of C6 and C8, subtracting them to the formation of the C5b67 complex [24]. An interesting role of C1 was proposed by B. Bode et al. [25], which indicated a relationship between Cq1 and ceramide transferase with the trafficking of ceramide and apoptosis. In this study, Cq1 appears to prevent autoimmunity and maintain self-tolerance by supporting the efficient clearance of apoptotic cells. In AD, the acute phase proteins are counter-regulated compared to iNPH as a specific fragment of complement and of specific immunoglobulins.

In iNPH, proteins involved in synaptic signaling and axogenesis, including substrates BACE1, APP, SEZ6L and SEZ6L2, were statistically lower, as were secretory proteins, glycosylation proteins and proteins involved in lipid metabolism (APOH and LCAT). Conversely, the inhibitor of metalloproteinase (TIMP1) and afamin (AFM) increased. The latter is a human vitamin E-binding protein that counteracts calcium release and oxidative stress when increased [26]. Collectively, these data suggest that in iNPH, the neurodegenerative pattern typical of AD is absent, and this is possibly associated with the increment of very long chain Cers that may exert a protective role. Thus monitoring very long chain Cers and specific markers like C6, C8, APP, SEZ6L, SEZ6L2, BACE1, AFM and TIMP1 could easily differentiate these two disorders.

What about the possible evolution towards AD? IPA pathway analysis indicated that inflammation is present but not neurodegeneration. However, comparing iNPH and C, this pattern is slightly increased, suggesting that a persisting inflammation can promote the evolution toward a neurodegenerative status [27]. The LXR/RXR pathway, which links metabolism and lipid transport, is also activated. These results indicate that a set of proteins and very long chains Cers (C22:0, C24:0) can predict the evolution toward AD becoming targets for its prevention. Furthermore, high levels of complement components C8 and C9 and of specific immunoglobulins together with a high level of the calcium phosphate-binding protein fetuin-B are a trait of AD.

From our results, several markers already described in other studies are confirmed, in particular, APP, which was recently suggested to be included in the panel of markers confirmatory for iNPH [10]. Although with a number of limitations, the present study provides hints particularly for monitoring the complement cascade and acute phase proteins and very long chain Cers as diagnostic markers of iNPH. Complement components C8, C9 and fetuin-B, together with NCAN and BCAN, should be monitored to predict the evolution toward AD, with these proteins being involved in neurodegeneration. While the relationship between sphingolipid dysregulation was established in AD, there is still uncertainty regarding the exact lipid species that are directly involved in neurodegeneration. It was collectively established that accumulation of Cer C16, C18 and C20 is a trait of AD patients [28,29,30,31]; however, the presence of very long chains Cers (C22 and C24) was observed for the first time in iNPH only and appeared to characterize this disorder. It is tempting to associate this feature to a protective role of these species toward APP and BACE1, inflammation, ER stress and ROS generation supported by proteomic data and from the literature. We can speculate about a relationship between specific protein differential levels and Cers levels and the protective role of very long chains Cers to counteract ER stress, mitochondrial damage and ROS generation [32]. Furthermore, long chains sphingolipids can also predict the evolution toward AD since a change in the pattern distribution of Cers can cause changes in the lipid raft [33] and a loss of the protective role of very long chain Cers.

## 4. Materials and Methods

### 4.1. Patients

This study involved 24 iNPH patients (12 women and 12 men), 18 AD patients (9 women and 9 men) and 18 aged subjects with cognitive integrity (C) (9 women and 9 men) enrolled at the Fondazione Ca’ Granda, IRCCS Ospedale Maggiore Policlinico, Milan, Italy (Table 1 and Supplementary Table S4). The present study conforms to the principles of the Helsinki Declaration, and the study protocol received approval from the Ethical Committee of Fondazione Ca’ Granda IRCCS Ospedale Maggiore Policlinico, Milan, Italy. Informed consent was obtained from either patients or their legal representatives. The inclusion criteria for the study were: the presence of anamnestic, clinical and neuroimaging criteria suggestive for probable iNPH according to the 2005 International Guidelines and ineligibility for shunting surgery [34]. All patients underwent a CSF tap evaluation including Barthel index from which the Barthel Continence Index (BCI), Mini-Mental State Examination (MMSE), Tinetti scale, and Timed Up-and-Go (TUG) test. Before and after CSF tap, a brain computed tomography or magnetic resonance was performed to confirm the ventricular enlargement (i.e., Evan index > 0.31) and no macroscopic obstruction to CSF flow as described in Rossi et al. [35]. The exclusion criteria were: secondary hydrocephalus, MMSE score <20/30 or gait disorders secondary to other evident causes [35]. Concerning controls, they underwent a comprehensive geriatric assessment that includes medical history, physical and neurological examination, neurocognitive evaluation (MMSE), computed tomography or MRI scan to exclude the presence of neurological and/or cognitive disorders. Concerning laboratory tests, the assessment of levels of tau, phospho-tau (*p*-tau) and amyloid-β (Aβ) proteins by ELISA (Innogenetics SRL, Pomezia, Italy) were performed. The diagnosis of AD was made according to current recommendations [36]. CSF samples were drawn in polypropylene tubes after a lumbar puncture at the L4/L5 or L3/L4 interspace, centrifuged at 4 °C and stored at −80 °C until analysis.

### 4.2. Lipid Extraction

Sphingolipids were extracted from CSF according to a previous study, with minor modification [37]. Briefly, 0.1 mL of CSF was mixed with 0.1 mL of ultrapure water and 1.5 mL of methanol/chloroform 2:1, and fortified with internal standards 100 pmol: ceramide (d18:1/12:0), sphingomyelin (d18:1/12:0), and 10 pmol of glucosyl (β)ceramide (d18:1/12:0). Samples were briefly sonicated and heated at 48 °C overnight. Then, 0.15 mL of potassium hydroxide (KOH) 1 M in methanol was added to every sample, and after 2-h incubation at 37 °C, the solution was neutralized with 0.15 mL of acetic acid 1 M and dried with Speedvac. Samples were then resuspended in 0.5 mL of methanol and transferred to a clean Eppendorf tube. Samples were dried, resuspended in 0.15 mL of methanol, and centrifuged for 3 min at 10,000× *g*. Liquid phases were collected in UPLC glass vials and stored at −80 °C.

### 4.3. Multiple Reaction Monitoring MS (MRM-MS)

Ceramides, hexosylceramides, sphingomyelins and dihydrosphingomyelins were quantified using a Xevo TQ-S micro mass spectrometer (Waters, Milford, MA, USA). Sphingolipid extracts were injected and separated on a C8 Acquity UPLC BEH 100 mm × 2.1 mm id, 1.7 µm (Waters, Milford, MA, USA) kept at 30 °C, with the following gradient: 0.0 min—80% B; 3 min—90% B; 6 min—90% B; 15 min—99% B; 18 min—99% B; 20 min—80% B, at 0.3 mL/min flow rate. Phase B consisted of 1 mM ammonium formate in methanol, 0.05 mM formic acid, while phase A was 2 mM ammonium formate in H_2_O, with 0.05 mM formic acid. An electrospray interface operating in positive ion mode was employed to obtain MS/MS spectra by acquiring MRM transitions of Cers (cone voltage 46 V, collision energy 26 eV), HexCers (cone voltage 22 V, collision energy 40 eV) and SMs and DhSMs (cone voltage 54 V, collision energy 28 eV).

The capillary voltage was set at 3.5 kV. The source temperature was set to 150 °C. The desolvation gas flow was set to 1000, and the desolvation temperature was set to 350 °C. The data were acquired by MassLynx™ 4.2 (Waters, Milford, MA, USA) software and quantified by TargetLynx software (Waters, Milford, MA, USA).

### 4.4. Sample Preparation for Bottom-Up Proteomics

The CSF were randomly pooled using the same protein amount into 18 sub-pools (6 sub-pools for iNPH patients, 6 sub-pools for AD patients and 6 sub-pools for healthy cognitive subjects), homogeneous for biometric parameters and sex-based (3 sub-pools for women and 3 for men for each condition). The sub-pooling was necessary since, for ethical reasons, the availability of CSF was minute, and this approach enables a reduction in the variance among biological groups, thereby increasing the power to detect changes when few samples are available, and the variance is high [38,39]. One hundred microliters of CSF were mixed with the same volume of 0.2% RapiGest (Waters, Milford, MA, USA). Dithiothreitol (DTT) was added to a final concentration of 5 mM for cysteine reduction, and samples were incubated for 30 min at 60 °C. Iodoacetamide (IAA) was added to a final concentration of 15 mM and incubated for 30 min in the dark. Proteins were digested with trypsin (Promega Italia SRL, Milano Italy) using an enzyme–protein ratio of 1:50 at 37 °C overnight. The digestion was conducted for 24 h, and RapiGest precipitated by adding trifluoroacetic acid (TFA) to a final concentration of 0.5% and samples incubated for 45 min at 37 °C. After centrifugation at 13,000 rpm for 10 min, the supernatants were recovered, and the peptide concentration was determined by Pierce™ Quantitative Colorimetric Peptide Assay (Thermo Scientific, Waltham, Massachussetts, USA).

### 4.5. Liquid Chromatography with Tandem Mass Spectrometry (LC–MS/MS)

An LC–ESI–MS/MS analysis was performed on a Dionex UltiMate 3000 HPLC System with an Easy Spray PepMap RSLC C18 column (150 mm, internal diameter of 75 μm; Thermo Scientific, Waltham, MA, USA). Gradient: 5% ACN in 0.1% formic acid for 10 min, 5–35% ACN in 0.1% formic acid for 139 min, 35–60% ACN in 0.1% formic for 40 min, 60–100% ACN for 1 min, 100% ACN for 10 min at a flow rate of 0.3 μL/min. The eluate was electrosprayed into an Orbitrap Tribrid Fusion (Thermo Fisher Scientific, Bremen, Germany) through a nanoelectrospray ion source (Thermo Fisher Scientific Bremen, Germany,). The LTQ-Orbitrap was operated in positive mode in data-dependent acquisition mode to automatically alternate between a full scan (350–2000 *m*/*z*) in the Orbitrap (at resolution 60,000, AGC target 1,000,000) and subsequent CID MS/MS in the linear ion trap of the 20 most intense peaks from full scan (normalized collision energy of 35%, 10 ms activation). Isolation window: 3 Da, unassigned charge states: rejected, charge state 1: rejected, charge states 2+, 3+, 4+: not rejected; dynamic exclusion enabled (60 s, exclusion list size: 200). Mass spectra were analyzed using MaxQuant software (version 1.6.3.3). The initial maximum allowed mass deviation was set to 6 ppm for monoisotopic precursor ions and 0.5 Da for MS/MS peaks. Enzyme specificity was set to trypsin/P, and a maximum of two missed cleavages was allowed. Carbamidomethylation was set as a fixed modification, while N-terminal acetylation and methionine oxidation were set as variable modifications. The spectra were searched by the Andromeda search engine against the Homo Sapiens Uniprot sequence database (release 15.01.2020). Protein identification required at least one unique or razor peptide per protein group. Quantification in MaxQuant was performed using the built-in XIC-based label-free quantification (LFQ) algorithm using fast LFQ. The required false positive rate (FDR) was set to 1% at the peptide, 1% at the protein and 1% at the site-modification level, and the minimum required peptide length was set to 7 amino acids.

### 4.6. Statistical and Bioinformatic Analysis

Age range and levels of Aβ, tau and p-tau proteins were compared between groups using a one-way ANOVA with Bonferroni’s correction if data were normally distributed, or with Dunn’s correction if they were not, using SigmaPlot software version 12.0. A statistical analysis on sphingolipid data was conducted using Origin 2019 software (Adalta; normality test, Kruskal–Wallis ANOVA with a post-hoc test), and boxplots were generated by the same software. Statistical analyses on LFQ data were conducted using one way-ANOVA in Perseus software (version 1.6.1.3). Only proteins present and quantified in at least 80% of technical and biological repeats were considered as positively identified in a sample and used for statistical analyses. A post-hoc test (Permutation-based FDR < 0.05) was carried out to identify proteins differentially expressed among different conditions. Bioinformatic analysis was carried out by Ingenuity Pathway Analysis (IPA^®^; QIAGEN Bioinformatics, Redwood City, CA, USA). The quantitative protein data were imported into IPA software to identify protein–protein interactions, canonical pathways, disease and biofunctions most strongly associated with the protein list [40]. ROC curves and AUC values were calculated with MetaboAnalyst software 5.0.

## 5. Limitations

The authors are aware of the limitation of the present study, which is the relatively small number of available samples. We cannot exclude that our findings might have been driven by third factors (i.e., specific characteristics of the recruited population, particularly the age of iNPH), which are not considered in the present analyses. A verification study in an independent set of samples is currently being carried out, including subjects age-matched and centenarians to confirm the observed changes in men and women by MRM-based MS analysis of CSF and serum.

## 6. Conclusions

The novelty introduced by this study is the identification of specific very long chains Cers and of a reasonable number of proteins able to implement the set of markers for a more precise diagnosis of iNPH. Importantly, the results provide a set of molecules to be monitored to follow up patients who are at risk of evolving toward AD.

## Figures and Tables

**Table 1:** Participants’ characteristics.

	Aged Subjects with Cognitive Integrity, *n* = 18	iNPH Patients, *n* = 24	AD Patients, *n* = 18
Age, years, median Age, years, min-max Age, years standard deviation (s.d.)	73 60–84 ±7.6	86 73–91 ±5.6	76 70–82 ±3.5
Gender, (M)	9	12	9
Aβ, pg/mL,median Aβ, pg/mL, min-max Aβ, pg/mL, s.d.	1223.5 457–1515 ±298	777.5 266–1248 ±232	433.5 226–654 ±117.5
Tau, pg/mL,median Tau, pg/mL, min-max Tau, pg/mL s.d.	153.5 54–420 ±98.6	157.5 46–676 ±150	627.5 110–2952 ±671.3
p-Tau, pg/mL, median p-Tau, pg/mL, min-max p-Tau, pg/mL, s.d.	35 7–61 ±13.8	31 15–73 ±13.7	79 22–475 ±99

**Table 2:** AUC values for Cers, HexCers and SMs species.

Name	AUC iNPH-C	AUC AD-C	AUC AD-iNPH
SM (d18:1/24:2)	1	0.99383	0.58449
Cer (d18:1/20:1)	0.99769	0.61728	0.89815
Cer (d18:1/16:1)	0.96528	0.60802	0.94444
Cer (d18:1/20:0)	0.90509	0.62346	0.90278
Cer (d18:1/24:0)	0.90046	0.50309	0.97685
SM (d18:1/24:1)	0.875	1	0.5787
Cer (d18:1/24:1)	0.84722	0.62963	0.91204
Cer (d18:1/22:0)	0.80787	0.51235	0.86574
Cer (d18:1/18:0)	0.77546	0.62037	0.65741
Cer (d18:1/18:1)	0.75	0.60802	0.72222
HexCer (d18:1/24:2)	0.72917	0.50617	0.7338
HexCer (d18:1/24:1)	0.72685	0.53086	0.7662
SM (d18:1/18:0)	0.71412	0.54321	0.62731
HexCer (d18:1/24:0)	0.69907	0.66049	0.81019
HexCer (d18:1/16:0)	0.65278	0.53395	0.6875
Cer (d18:1/16:0)	0.64352	0.58642	0.72222
HexCer (d18:1/22:0)	0.64352	0.5	0.67593
SM (d18:1/20:0)	0.6412	0.64352	0.52199
HexCer (d18:1/20:0)	0.63194	0.55247	0.6713
HexCer (d18:1/18:0)	0.61111	0.53395	0.68056
SM (d18:1/14:0)	0.58449	0.50617	0.60532
SM (d18:1/22:1)	0.53819	0.60494	0.53704
SM (d18:1/18:1)	0.53588	0.55093	0.58912
SM (d18:1/20:1)	0.53241	0.54784	0.50347
SM (d18:1/22:0)	0.51273	0.60648	0.55671
SM (d18:1/24:0)	0.51273	0.60802	0.55787
SM (d18:1/16:1)	0.50116	0.50926	0.54745
SM (d18:1/16:0)	0.5	0.5	0.5625

**Table 3:** AUC values for proteins with an opposite trend in iNPH and AD.

T: Gene Names	AUC iNPH-C	AUC AD-C	AUC iNPH-AD
A1BG	NA	0.86574	0.91667
AFM	0.72222	1	0.95312
ALDOA	0.52431	0.50463	0.5
APLP1	0.90278	0.5787	0.80729
APLP2	0.625	1	0.92708
APOE	0.66319	0.67593	0.54167
APOH	0.87847	0.65278	0.79167
APP	0.79167	0.94444	0.84896
BTD	0.79514	0.73611	0.61979
C16orf89	0.89236	0.54167	0.92708
C6	0.99653	0.88426	0.56771
C8G	0.61806	0.625	0.52604
CADM2	0.8125	0.91667	0.69792
CADM3	0.93403	0.87037	0.76562
CALR	0.82986	1	0.69792
CBLN3	0.95833	0.99074	0.60938
CFD	0.59722	1	0.94271
CFI	0.93056	0.81944	0.77083
CGREF1	0.91146	0.96296	0.54167
CHGA	0.98611	0.78241	0.90104
CHL1	0.82292	0.90278	0.96354
CLSTN1	0.9375	0.99074	0.67188
CLSTN3	0.78125	1	0.97396
CNTFR	0.84028	0.72222	0.55208
CNTN1	0.72569	1	1
CP	0.78125	0.95833	0.6901
CRTAC1	0.61111	0.91204	0.85417
DAG1	0.68403	0.72222	0.50521
DKK3	0.72222	0.99537	0.93229
FAT2	0.74306	0.5463	0.70312
FCGBP	0.84028	1	0.56771
FGA	0.59028	0.94907	0.94792
FGB	0.84722	0.96296	1
FGG	0.65104	0.9537	0.76562
FSTL4	0.71875	0.95833	0.8776
GOLM1	0.80903	0.79167	0.66667
GPR37L1	0.625	0.56944	0.54688
HABP2	0.65104	0.75	0.64062
HPX	0.59028	0.84259	0.80729
IDS	0.90278	0.79167	0.92188
IGHA1	0.94097	0.63889	0.94792
IGHG1	0.78472	1	0.88542
IGHG4	0.52431	0.64352	0.57812
IGHV3OR16-9	0.79514	0.97222	0.86979
IGKC	0.74653	0.74537	0.79167
IGKV3D-11	0.86458	0.98611	1
ITIH1	0.98264	1	0.51562
ITIH4	0.78125	1	0.99479
LCAT	0.71875	0.95833	0.8776
LRG1	0.8125	1	0.77083
LRRC4B	0.98264	1	0.66927
LY6H	0.70139	0.59259	0.52083
LYZ	0.65625	1	0.78125
MEGF8	0.58333	1	1
NEGR1	0.83681	0.97685	0.79688
NELL2	0.55903	0.83796	0.89062
NEO1	0.55903	1	1
NOV	0.78472	0.77778	0.65104
NPTX1	0.87326	1	0.59375
NRCAM	0.84722	0.51852	0.84896
NRXN1	0.62153	0.78241	0.51042
NRXN2	0.94097	1	0.83333
NRXN3	0.74306	0.98611	1
OPCML		0.9213	0.75
ORM1	0.85417	0.88426	0.51042
ORM2	0.71528	0.73611	1
PAM	0.78472	0.5463	0.78646
PCDH1	0.59722	0.74537	0.79167
PIK3IP1	1	1	0.5
POMGNT1	0.56076	0.90509	0.9401
QPCT	0.80208	0.98611	0.63021
RELN	0.71875	1	0.85417
SCG3	0.77431	1	1
SERPINA1	0.75347	0.57407	0.8125
SERPINA3	0.94444	0.68056	0.77083
SERPINI1	0.53472	1	1
SEZ6L	0.71875	0.91667	0.83854
SEZ6L2	0.71528	0.9213	0.76042
SIRPA;SIRPB1	0.67014	0.76389	0.60417
SLITRK1	0.62153	0.77315	0.67448
SPOCK1	0.73611	0.79167	0.89062
TIMP1	0.71875	0.93519	0.95312
VASN	0.58333	0.97685	0.97396
VGF	0.88194	0.89352	0.97917

## Data Availability

The data presented in this study are available in the Supplementary Material. Study data from this human study other than those published in this work are under privacy regulations but can be obtained on a case-to-case basis upon reasonable request from the corresponding author.

## Supplementary Materials

The following are available online at https://www.mdpi.com/article/10.3390/ijms22158034/s1, Table S1: List of changed proteins with the opposite trend in iNPH and AD, Table S2: List of changed proteins with the same trend in iNPH and AD, Table S3: Ingenuity Pathway Analysis canonical pathways; Figure S1: Ingenuity Pathway Analysis networks, Figure S2 Sex distribution in box plots of hexosylceramide species, Table S4: Detailed participants’ characteristics.
