# Supplementary material for: Novel Insight in Idiopathic Normal Pressure Hydrocephalus (iNPH) Biomarker Discovery in CSF
Source: Int J Mol Sci. 2021 Jul 27;22(15):8034. doi: 10.3390/ijms22158034 (PMC8347603; doi:10.3390/ijms22158034)
Supplement: Supplementary file 1 [file ijms-22-08034-s001.zip › Supplementary Figures.pdf]

Supplementary Figures

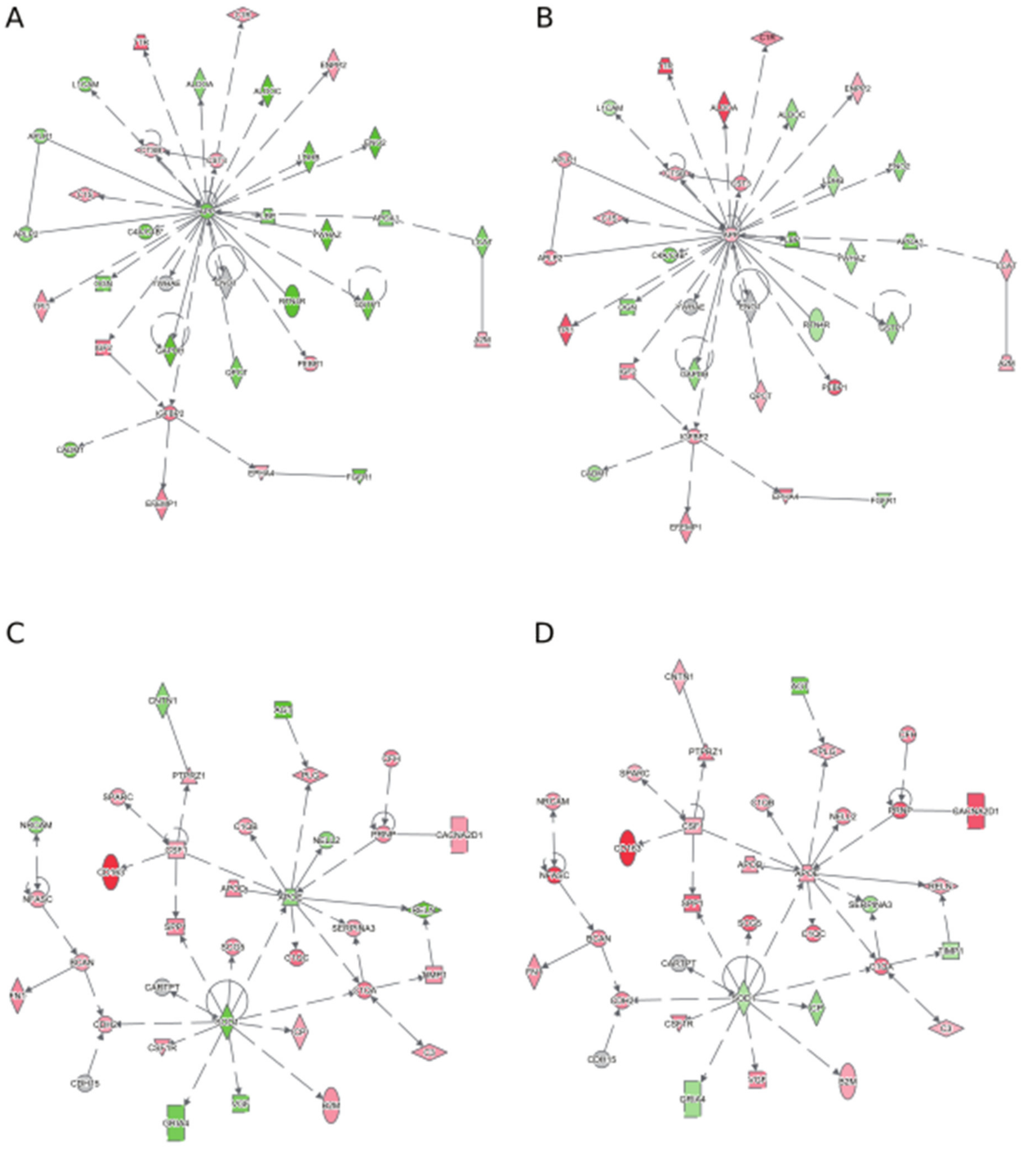

Figure S1. Ingenuity Pathway Analysis networks. The first network (**A**) iNPH vs controls; (**B**) AD vs controls, grouped 35 proteins mainly involved in neurological disease, scoring 56; the second (**C**) iNPH vs controls; (**D**) AD vs controls grouped 35 proteins involved in metabolic disease, scoring 56.

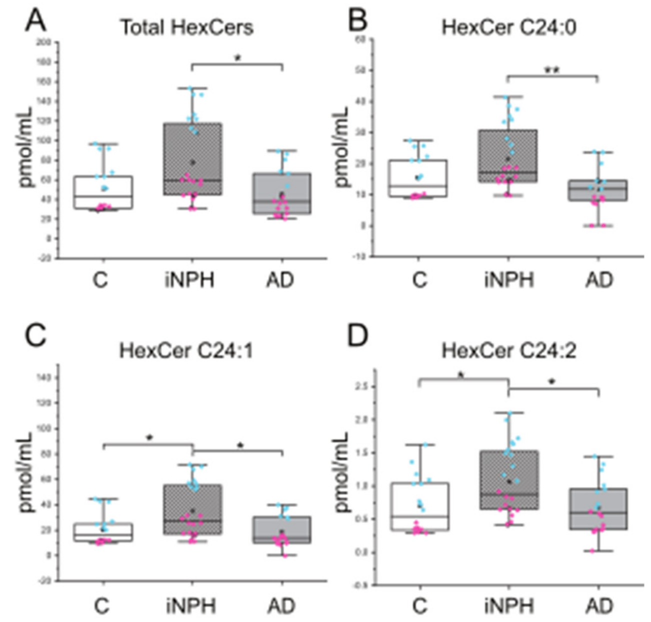

Figure S2. Sex distribution in box plots of CSF hexosylceramide species. Men are shown as blue circles whereas women are shown as pink circles. Data were analyzed using Kruskal-Wallis ANOVA, followed by Dunn's post hoc test for multiple comparisons. * p-value < 0.05, ** p-value < 0.01, *** p-value<0.001.
